# Selective inhibition mechanism of RVX-208 to the second bromodomain of bromo and extraterminal proteins: insight from microsecond molecular dynamics simulations

**DOI:** 10.1038/s41598-017-08909-8

**Published:** 2017-08-18

**Authors:** Qianqian Wang, Ying Li, Jiahui Xu, Yuwei Wang, Elaine Lai-Han Leung, Liang Liu, Xiaojun Yao

**Affiliations:** State Key Laboratory of Quality Research in Chinese Medicine, Macau Institute for Applied Research in Medicine and Health, Macau University of Science and Technology, Taipa, Macau China

## Abstract

RVX-208 is a recently reported inhibitor of bromo and extraterminal (BET) family proteins (including BRD2-4 and BRDT) with selectivity for the second bromodomain (BD2), currently in phase III clinical trials. Despite of its promising antitumor activity, due to the conserved folds of the first and second bromodomains (BD1 and BD2), the detailed selectivity mechanism of RVX-208 towards BD2 over BD1 is still unknown. To elucidate selective inhibition mechanism of RVX-208 to BD2, microsecond molecular dynamics simulations were performed in this study for BRD2-BD1, BRD2-BD2 and BRD4-BD1 with and without RVX-208, respectively. Binding free energy calculations show that there exists strongest interaction between RVX-208 and BRD2-BD2. Leu383 and Asn429 are two most important residues of BRD2-BD2 for binding to RVX-208. Structural network analysis reveals that RVX-208 can shorten the communication path of ZA and BC loops in BRD2-BD2 pocket, making pocket more suitable to accommodate RVX-208. Additionally, different behaviors of His433 (Asp160 in BRD2-BD1) and Val435 (Ile162 in BRD2-BD1) in BRD2-BD2 are key factors responsible for selective binding of RVX-208 to BRD2-BD2. The proposed selective inhibition mechanism of RVX-208 to BRD2-BD2 can be helpful for rational design of novel selective inhibitors of the second bromodomain of BET family proteins.

## Introduction

Bromodomains (BRDs) are protein modulators that specifically recognize acetylated lysine-containing sequences as an “epigenetic reader”. To date, 61 different BRDs from 46 nuclear and cytoplasmic proteins were discovered and could be divided into eight families based on their sequence and structural similarity^1, 2^. Despite sequence diversity, all BRD modules share a conserved fold comprised by a four-helix bundle (αZ, αA, αB and αC), linked by ZA and BC loops that contribute to substrate specificity^3^. Cocrystal structures with peptide substrates demonstrated that the acetylated lysine was recognized by a central hydrophobic cavity and anchored by hydrogen bonds to an asparagine residue present in most BRDs^1, 4–6^. With acetylation motifs often found in macromolecular complexes implicated in DNA repair, chromatin remodeling and cell-cycle control^7–9^, the architecture of acetyl-lysine pockets of BRDs makes them attractive targets for the design of potent inhibitors^10–14^.

The bromo and extraterminal (BET, including BRD2-4 and BRDT) proteins, as transcriptional regulators, are closely associated with the occurring and development of cancers such as lung cancer^15^ and NUT midline carcinoma^16^. Inhibiting the recognition interaction between bromodomain and acetyl-lysine by small molecules is considered as an effective approach to halt tumor development. Over the past decade, many diverse inhibitors of BET proteins have exhibited significant antitumor activity^16–20^ and five of them (namely RVX-208^21^, I-BET762^22^, OTX015^23^, CPI-0610^24^ and TEN-010^25^) have entered clinical trials. But the problem is that all the inhibitors reported to date were multi-target or multi-domain except for RVX-208. Structural analyses show that not only all four BET proteins but also two homologous bromodomains (BD1/2) of each protein are highly conserved^1^, and selective inhibition of either BD1 or BD2 can result in distinct transcriptional outcomes^21, 26–28^. For instance, BD1-selective inhibition by olinone was shown to promote oligodendrocyte differentiation, but which did not occur upon inhibition of both domains^27^. The domain-specific inhibitors against BETs are highly needed to avoid adverse effects of prolonged pan-BET inhibition.

RVX-208 was a domain-selective inhibitor reported recently, with IC_50_ of 510 nM for BD2 and 170-fold lower than that to BD1^21^. As the first selective BRD-BD2 inhibitor, RVX-208 is currently undergoing phase III clinical trials for treating the cardiovascular disease, but the potential molecular mechanism of RVX-208 selectively inhibiting BD2 is still unclear. Although the development of computational methods especially molecular dynamics (MD) simulations makes the explanation of drug selectivity mechanism possible^29–31^, a crucial factor affecting result accuracy is the simulation timescale, as emphasized by Shaw *et al*.^32–34^. By the unbiased MD simulations, Hou *et al*.^35^ also revealed that structural characteristics provided by the microsecond timescale were distinct from that by nanosecond simulations. Therefore, in order to observe the dynamic characteristics of protein more reliable, long time simulation is greatly needed. Microsecond molecular dynamics simulations were performed in this study to investigate the binding modes of RVX-208 with bromodomain of BET proteins and to elucidate the selective inhibition mechanism of RVX-208 to the second bromodomain of bromo and extraterminal proteins.

## Results and Discussion

### Convergence assessment

**Figure 1:**
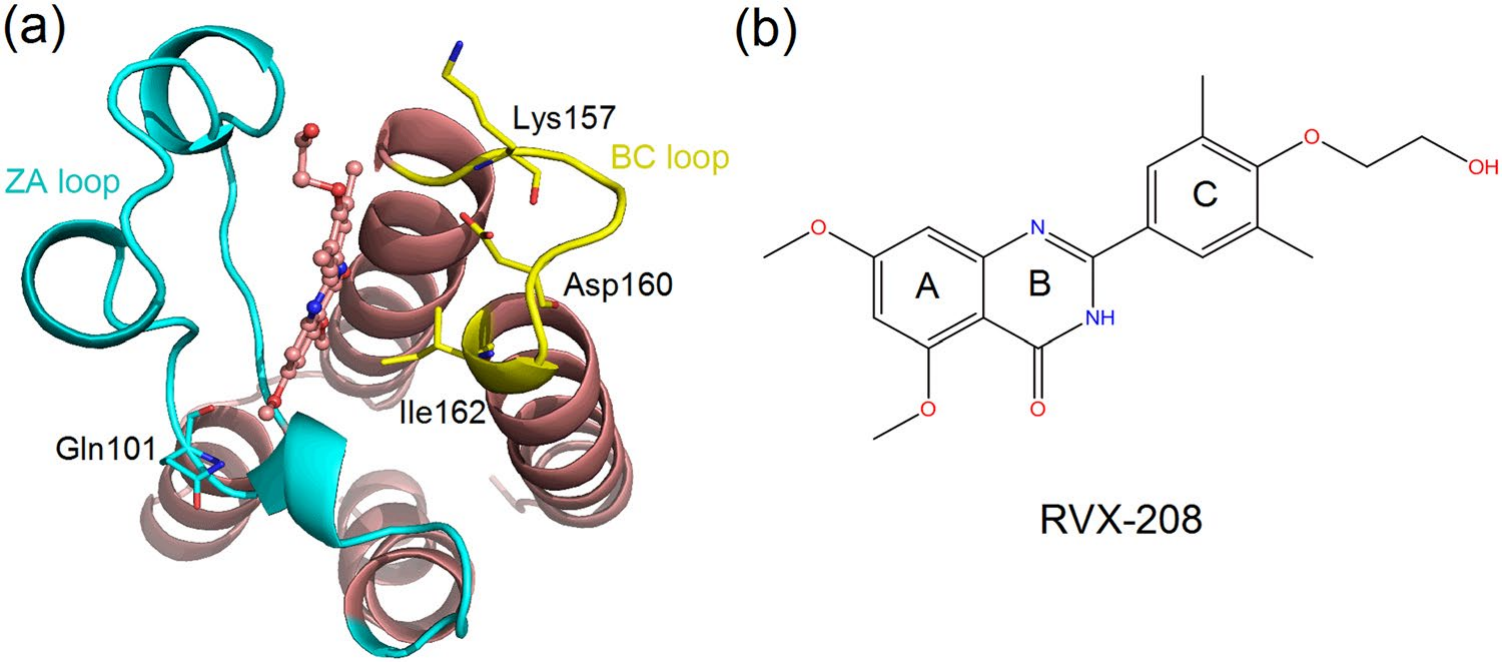
(**a**) The constructed complex structure of RVX-208 with BRD2-BBD1 and (**b**) The molecular structure of RVX-208. Blue and yellow cartoon regions represent ZA and BC loops, respectively. Residues in stick model are BRD2-BD1-specific in the pocket.

**Figure 2:**
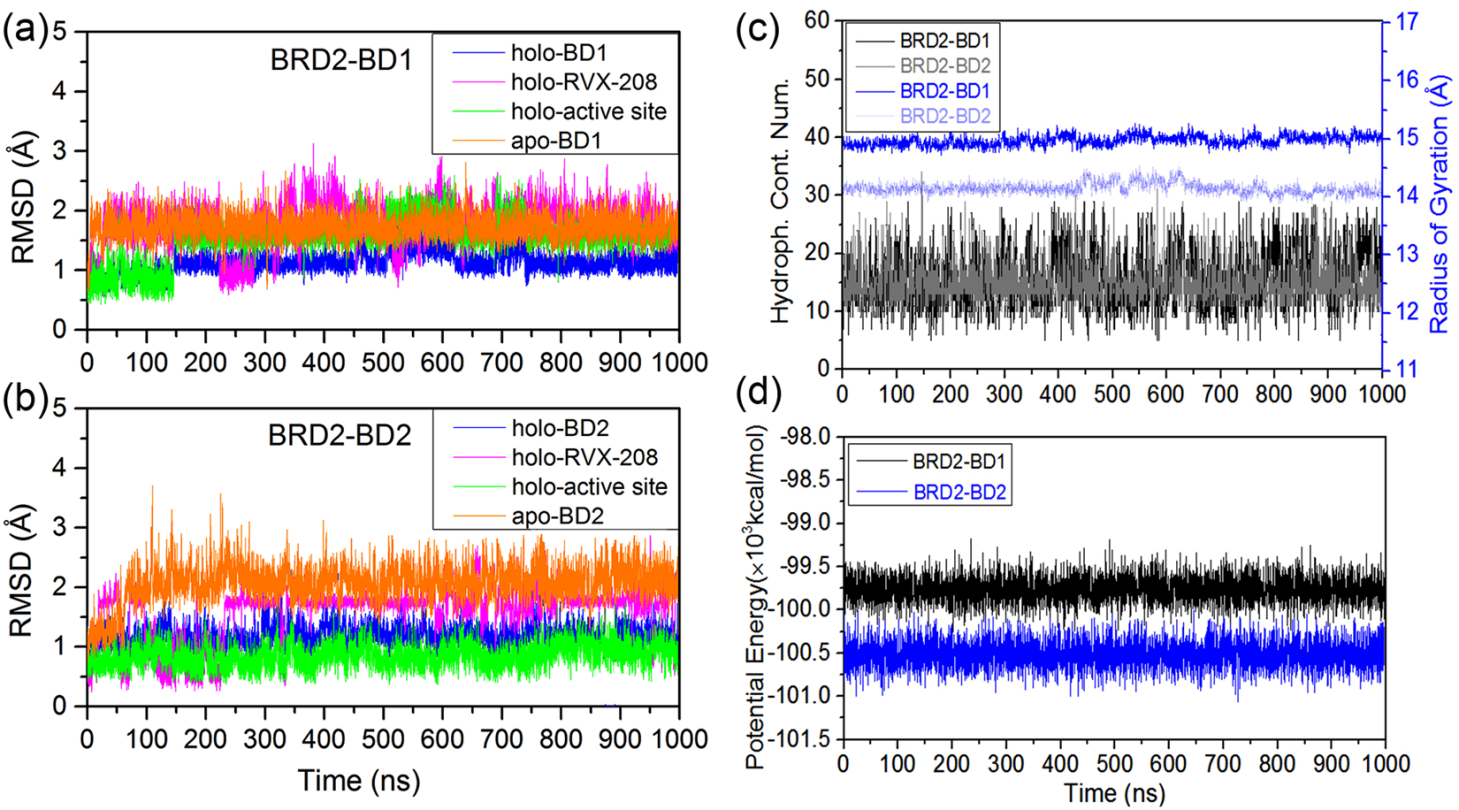
Time series of RMSDs of p-rotein, RVX-208 and active site in holo and apo systems of (**a**) BRD2-BD1 and (**b**) BRD2-BD2; time series of (**c**, left) contact number between CA atoms of hydrophobic residues of BDs and RVX-208, (c, right) radius of gyration of BDs and (**d**) potential energy in two complex systems.

Six systems were simulated totally, namely three holo-BDs (BRD2-BD1-RVX-208, BRD2-BD2-RVX-208 and BRD4-BD1-RVX-208) and three apo-BDs (BRD2-BD1, BRD2-BD2 and BRD4-BD1). The constructed complex of BRD2-BD1-RVX-208 and the structure of RVX-208 were shown in Fig. 1. To ensure results reproducible and more convincing, one additional parallel trajectory was also simulated for each complex (Table 1). Because we mainly aim to reveal the selective mechanism of RVX-208 for BD2 against BD1, BRD2-BD1s and BRD2-BD2s with/without RVX-208 were analyzed in more detail. Firstly, with respect to the starting structure, root-mean-square deviations (RMSDs) of protein CA atoms, heavy atoms of RVX-208, CA atoms of active site in holo and apo systems of BRD2-BD1 and BRD2-BD2 were monitored to assess the overall stability of simulations. From Fig. 2a and b, RMSDs of four systems fluctuate dramatically initially, and then remain stable from 600 ns especially for the last 200 ns, indicating the trajectory convergence. Usually, small molecules exert their inhibitory activity by binding to hydrophobic pocket of bromodomains to compete with lysine-acetyl substrate^18^. The calculated contact number of RVX-208 with hydrophobic residues of BD1 and BD2 of BRD2 almost keeps constant during the simulation (Fig. 2c, left). Furthermore, two other variables were also monitored and compared, namely radius of gyration for BDs (Fig. 2c, right) and potential energy (Fig. 2d) of BRD2-BD1(2)-RVX-208 complex systems. For simplification, the former was merged to hydrophobic contact result (Fig. 2c). The values of radius of gyration for holo-BD2 and potential energy for BRD2-BD2-RVX-208 system were both lower than that for BRD2-BD1-RVX-208 system, indicating that holo-BD2 protein was more compact and the corresponding complex system was more stable. The parallel three complex systems gave similar convergence trends (Fig. S1). Overall, each system converged from 600 ns, and the last 200 ns trajectory was used for the following analyses.

**Table 1:** The simulation systems in this study.

Systems	PDB entry	BD-unique binding residues		System size	Simulation time	Simulation Number
		ZA loop	BC loop			
BD1(BRD2)/RVX-208	2YDW + 4MR4	Q101	K157, D160, I162	30,716 atoms	1 μs	2
BD1(BRD2)				27,937 atoms	1 μs	1
BD2(BRD2)/RVX-208	4MR6	K374	P430, H433, V435	29,291 atoms	1 μs	2
BD2(BRD2)				25,270 atoms	1 μs	1
BD1(BRD4)/RVX-208	4MR4	Q85	K141, D144, I146	32,939 atoms	1 μs	2
BD1(BRD4)				30,088 atoms	1 μs	1

(Note: The residue number used in each system is corresponding to that in RCSB Protein Data Bank).

### Energy origin of selective mechanism of RVX-208 for BRD2-BD2

Here, the binding free energy of BRD4-BD1 and RVX-208 was calculated. In Table 2, both Δ*G*_GB_ and Δ*G*_PB_ in BRD2-BD2 system were lower than that in two BD1 systems (BRD2-BD1 and BRD4-BD1), suggesting stronger binding affinity of RVX-208 with BD2. The calculated binding free energies (−8.32 ± 0.09, −10.51 ± 0.16 and −10.02 ± 0.15 kcal/mol) between BDs and RVX-208 in three systems exhibit a consistent order with experimental results (−6.90, −8.47 and −7.84 kcal/mol)^21^. Among the individual terms, van der Waals interaction (Δ*E*_vdw_) predominates the total energy, while the nonpolar solvation term (Δ*G*_sol_np_PB_) contributes marginally to inhibitor binding. The overall electrostatics, however, disfavors complex formation. These results confirm that ligand association with poorly solvated binding pocket is dominantly driven by van der Waals interactions^36^. Considering the minor Δ*G*_sol_np_PB_ difference between BD1 and BD2 systems, from the perspective of energy, energetic origin of RVX-208 selective for BD2 is thus mainly from the higher Δ*E*_vdw_.

**Table 2:** The calculated binding free energy and its components (kcal/mol) of three complexes in two parallel trajectories.

Contributions	Run-1			Run-2		
	BRD2-BD1	BRD2-BD2	BRD4-BD1	BRD2-BD1	BRD2-BD2	BRD4-BD1
Δ*E*_ele_	−10.61 ± 0.31	−14.87 ± 0.29	−11.20 ± 0.15	−16.25 ± 0.72	−18.63 ± 0.80	−6.27 ± 0.24
Δ*E*_vdw_	−15.35 ± 0.35	−20.20 ± 0.55	−16.09 ± 0.62	−22.62 ± 0.43	−26.14 ± 0.55	−8.63 ± 0.44
Δ*E*_int_	0	0	0	0	0	0
Δ*E*_gas_	−25.96 ± 1.10	−35.07 ± 1.29	−27.30 ± 1.04	−38.87 ± 1.42	−44.77 ± 2.13	14.90 ± 0.32
Δ*G*_sol_np_GB_	−2.20 ± 0.04	−3.29 ± 0.06	−2.52 ± 0.08	−3.30 ± 0.19	−4.26 ± 0.20	−1.23 ± 0.09
Δ*G*_sol_polar_GB_	14.92 ± 0.52	19.63 ± 0.62	15.65 ± 0.44	23.27 ± 1.88	24.94 ± 2.05	8.95 ± 0.50
Δ*G*_sol_GB_	12.72 ± 0.29	16.34 ± 0.34	13.13 ± 0.28	19.97 ± 0.63	20.68 ± 0.65	7.72 ± 0.47
Δ*G*_polar_GB_	4.31 ± 0.09	4.75 ± 0.07	4.45 ± 0.10	7.02 ± 0.25	6.30 ± 0.12	2.60 ± 0.19
Δ*G*_np_GB_	−17.55 ± 0.50	−23.49 ± 0.44	−18.61 ± 0.32	−25.92 ± 0.48	−30.40 ± 0.59	9.86 ± 0.61
Δ*H*_GB_	−13.24	−18.73	−14.16	−18.90	−24.09	−7.25
−TΔ*S*	4.92 ± 0.08	8.22 ± 0.11	4.14 ± 0.09	12.64 ± 0.21	13.18 ± 0.33	2.17 ± 0.05
**Δ** ***G***_**GB**_	**−8**.**32** ± **0**.**09**	**−10**.**51** ± **0**.**16**	**−10**.**02** ± **0**.**15**	**−6**.**26** ± **0**.**10**	**−10**.**91** ± **0**.**21**	**−5**.**08** ± **0**.**09**
Δ*G*_sol_np_PB_	−2.21 ± 0.08	−3.29 ± 0.09	−2.52 ± 0.08	−3.30 ± 0.19	−4.26 ± 0.20	−1.23 ± 0.09
Δ*G*_sol_polar_PB_	16.16 ± 0.33	20.93 ± 0.67	16.77 ± 0.47	24.37 ± 2.02	26.39 ± 2.53	9.36 ± 0.34
Δ*G*_sol_PB_	13.95 ± 0.26	17.64 ± 0.52	14.26 ± 0.35	21.07 ± 1.38	22.13 ± 1.35	8.12 ± 0.30
Δ*G*_polar_PB_	5.55 ± 0.10	6.06 ± 0.15	5.57 ± 0.29	8.13 ± 0.27	7.75 ± 0.26	3.08 ± 0.15
Δ*G*_np_PB_	−17.56 ± 0.43	−23.46 ± 0.71	−18.61 ± 0.44	−25.92 ± 0.48	−30.40 ± 0.59	9.86 ± 0.61
Δ*H*_PB_	−12.01	−17.43	−13.05	−17.19	−22.65	−6.84
−TΔ*S*	4.92 ± 0.08	8.22 ± 0.11	4.14 ± 0.09	12.64 ± 0.21	13.18 ± 0.33	2.17 ± 0.05
**Δ** ***G***_**PB**_	**−7**.**09** ± **0**.**18**	**−9**.**21** ± **0**.**20**	**−8**.**91** ± **0**.**15**	**−4**.**55** ± **0**.**08**	**−9**.**47** ± **0**.**22**	**−4**.**67** ± **0**.**10**
**Δ** ***G***_**exp**_	**−6**.**90**	**−8**.**47**	**−7**.**84**	**−6**.**90**	**−8**.**47**	**−7**.**84**

**Figure 3:**
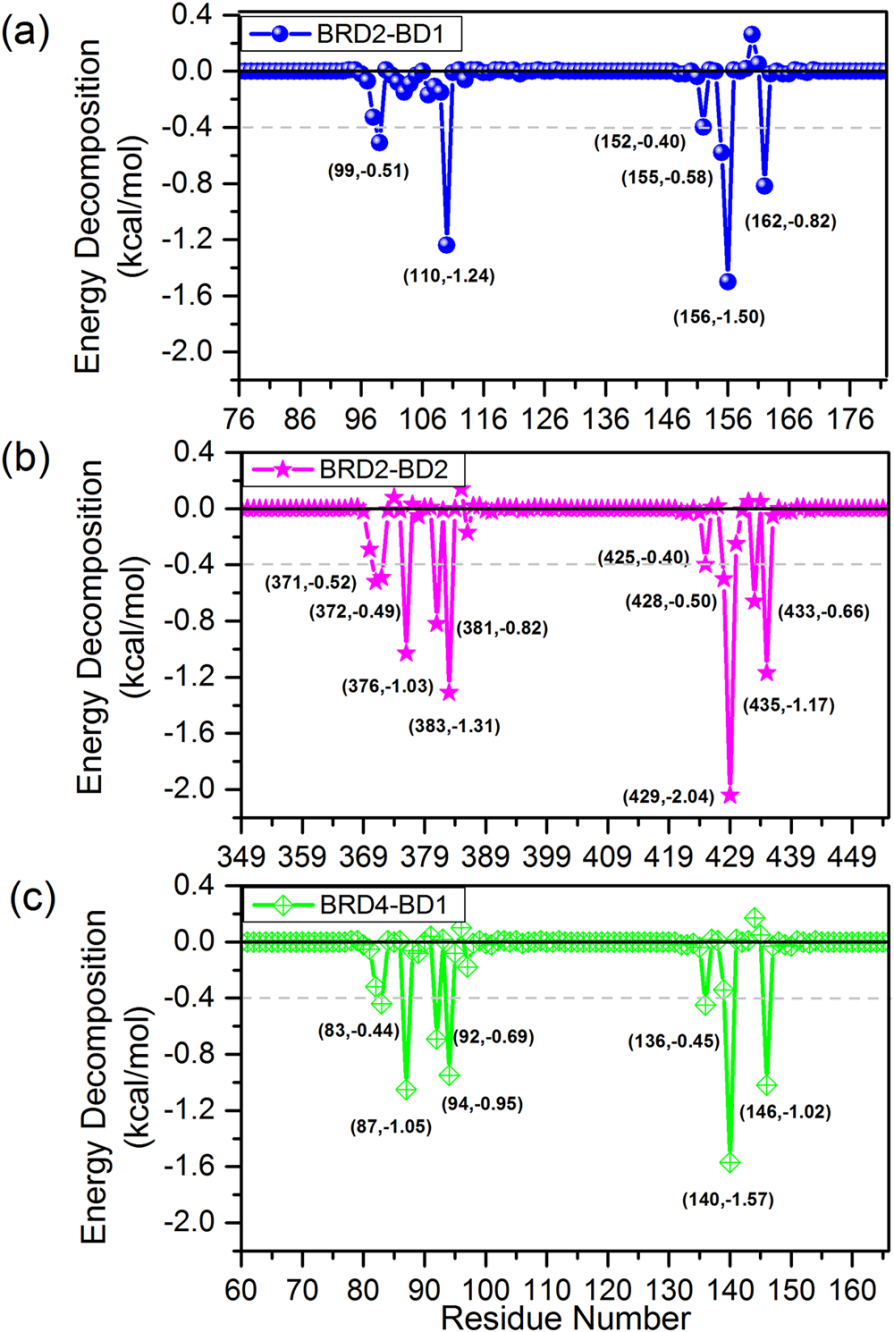
Per-residue energy decomposition in (**a**) BRD2-BD1, (**b**) BRD2-BD2 and (**c**) BRD4-BD1 systems. Residues with contribution more than 0.4 kcal/mol were labeled.

The total binding free energy was further decomposed into per-residue contribution of BD (Fig. 3 and Supplementary Fig. S2). The high consistency of per-residue interaction spectrum of BRD2-BD1 with BRD4-BD1 systems confirmed the reliability of our calculations (Figs 3a and c and S2a and c). Previous studies reported that the inhibitor-binding pocket on bromodomain was mainly composed of ZA and BC loops^14, 16^. From Figs 3 and S2, it is clear that, in three systems, residues with large contribution (>0.4 kcal/mol) locate in ZA and BC loops, and the two key residues were Leu110 and Asn156 in BRD2-BD1 system (corresponding to Leu383 and Asn429 in BRD2-BD2 system). By comparing these three systems, we can see that both the number and contribution of key residues of either ZA or BC loop in BRD2-BD2 system were higher than that in other two BD1 systems, suggesting the favorable binding of RVX-208 to BD2. Sequence alignment shows that four residues are different in inhibitor-binding pocket between BD1 and BD2, namely Gln101, Lys157, Asp160 and Ile162 (in BRD2-BD1 system, Table 1). In these four BD-specific residues, Ile162 is important for three systems, but His433 (Asp160 in BRD2-BD1 system) is also essential for BRD2-BD2 system. The effect of His433 during the binding of RVX-208 will be discussed in the following section.

### Structural network analyses of BRD2-BD1 and BRD2-BD2

**Figure 4:**
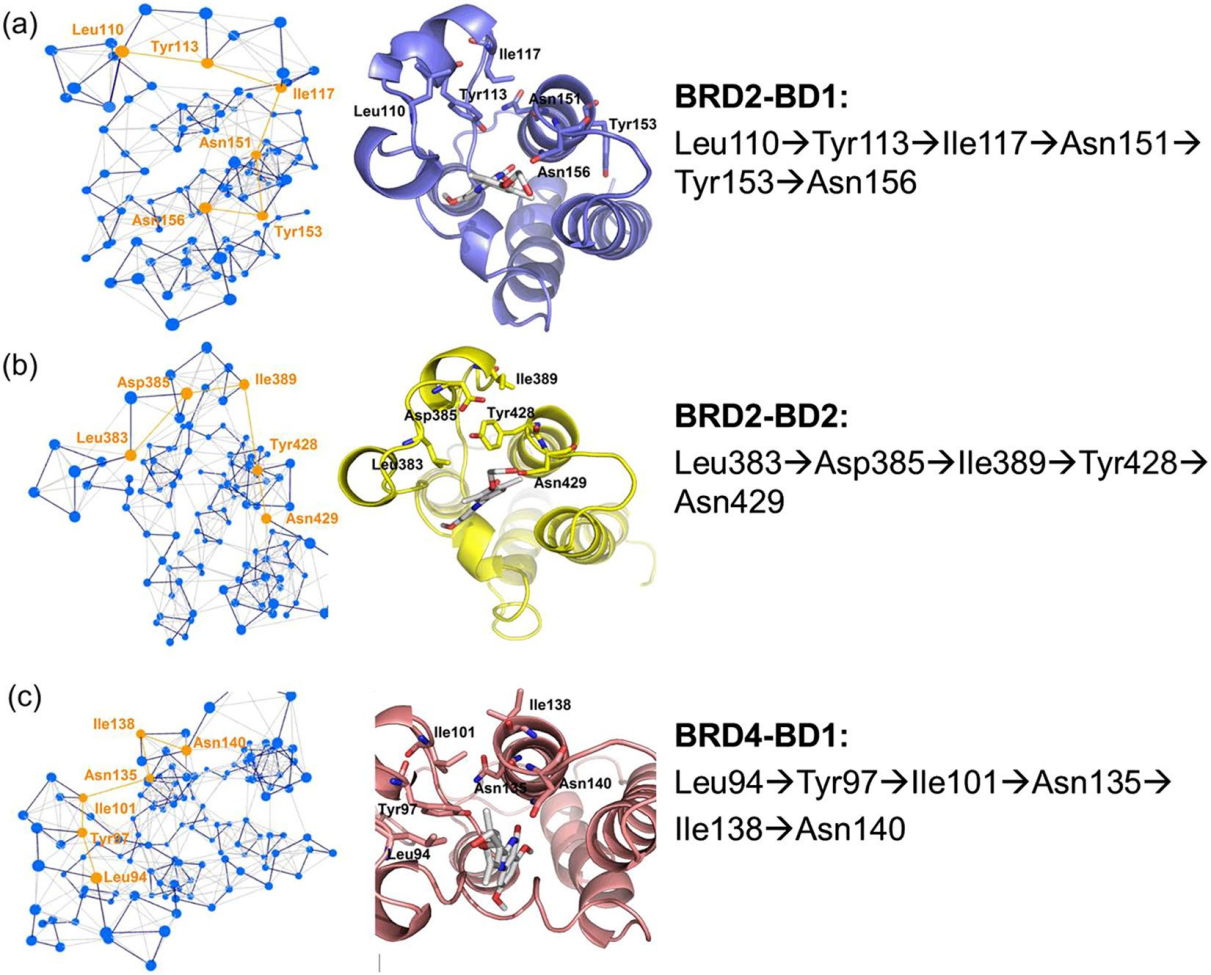
Shortest communication path between ZA and BC loops and the corresponding structure in (**a**) BRD2-BD1, (**b**) BRD2-BD2 and (**c**) BRD4-BD1 systems.

ZA and BC loops are two regions responsible for binding to RVX-208. However, they are not continuous in the sequence or structure, but distribute on both sides of RVX-208 in the space. To study how ZA and BC loops communicate in RVX-208-binding complex and how the communication affects RVX-208’s selectivity, two representative residues identified by energy decomposition, Leu110 (at ZA loop) and Asn156 (at BC loop), were used as terminal residues in the shortest path (Fig. 4). Figure 4a shows that the shortest path distance of communication between Leu110 and Asn156 in BRD2-BD1 system spanned four residues, equal to that in BRD4-BD1 system (Fig. 4c). Moreover, the involved residues in two BD1 systems (Tyr113, Ile117, Asn151 and Tyr153 in BRD2-BD1 system; Tyr97, Ile101, Asn135 and Ile138 in BRD4-BD1 system) were consistent except Tyr153 in BRD2-BD1 system. The parallel three complex trajectories showed the similar results (Fig. S3), suggesting the reproducibility of our simulations again. The comparison of shortest communication paths between BD1 and BD2 systems showed that the path in BD2 system involved three residues, less than that in BD1 systems. Furthermore, these residues were different from each other (Asp385, Ile389 and Tyr428 in BRD2-BD2 system). These results suggested that RVX-208 might alter or shorten the communication path of ZA and BC loops when binding to BD2, making pocket readjusted and more suitable for accommodating RVX-208. Considering the consistent behavior of two parallel trajectories for each complex, the following analyses were only performed on the trajectories of the first run.

### Differences of RVX-208 binding to BRD2-BD1 and BRD2-BD2

**Figure 5:**
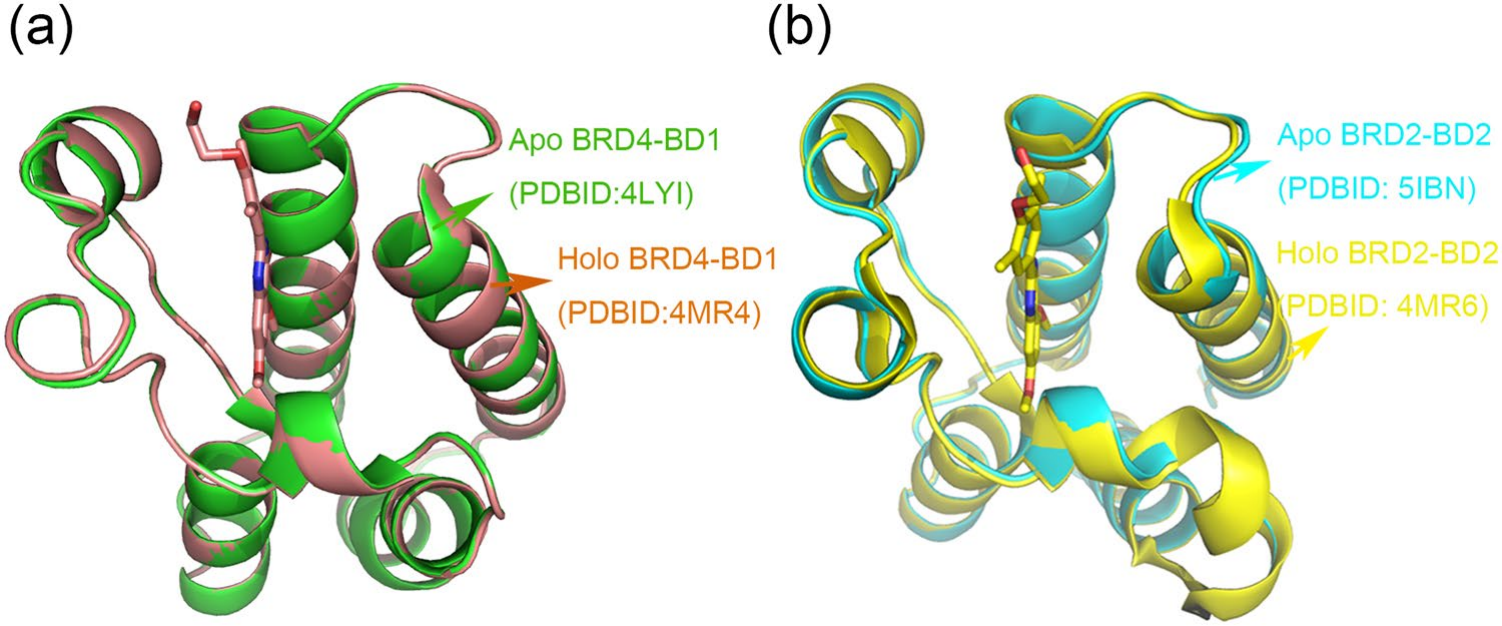
Crystal structural alignment of apo and holo BDs in (**a**) BRD4-BD1 and (**b**) BRD2-BD2 systems.

**Figure 6:**
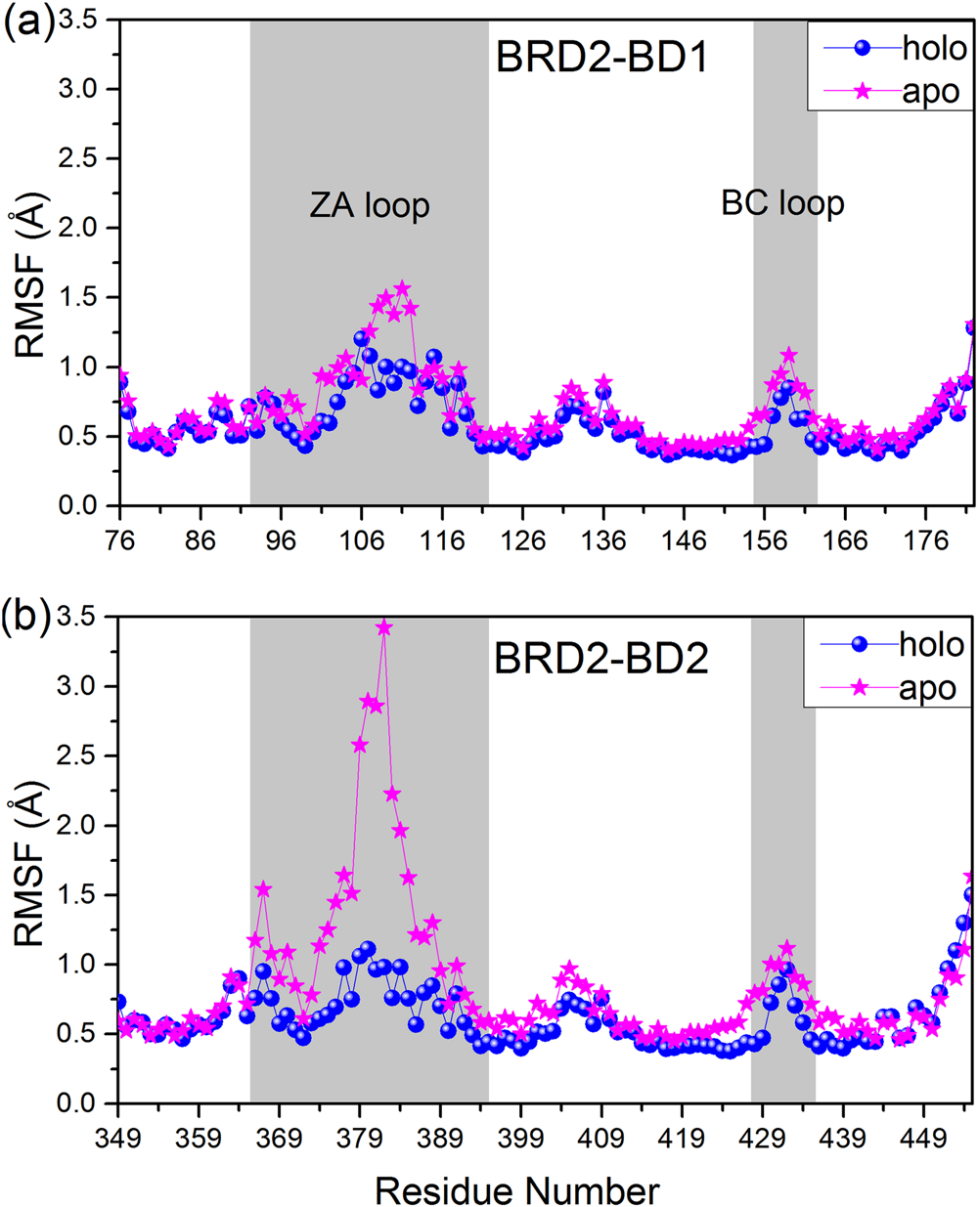
RMSFs as a function of residues in apo and holo systems of (**a**) BRD2-BD1 and (**b**) BRD2-BD2.

**Figure 7:**
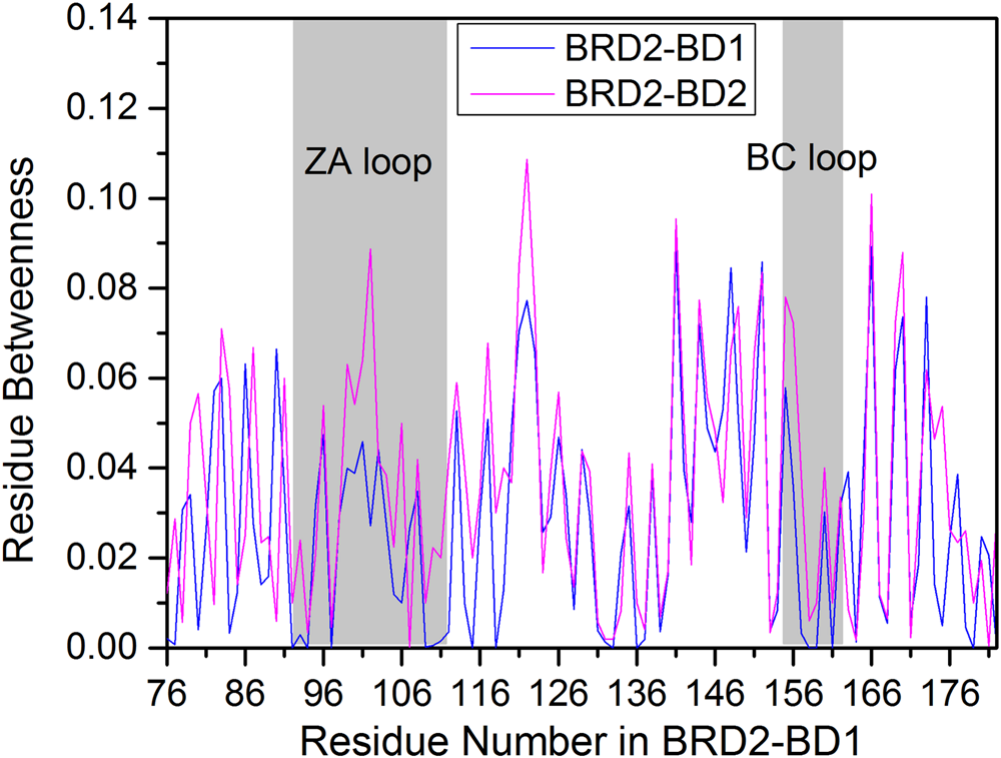
Residue betweenness as a function of residues in holo systems of BRD2-BD1 and BRD2-BD2. To exhibit betweenness difference of two systems clearly, residue number used in BRD2-BD2 system is corresponding to that of RCSB Protein Data Bank (PDB ID: 4MR4) in BRD2-BD1 system.

We firstly aligned crystal structures of apo-BD1 with holo-BD1, and apo-BD2 with holo-BD2 (Fig. 5). Herein, to eliminate the effect of manual construction on results, BRD4-BD1 system rather than BRD2-BD1 system was used. From Fig. 5, in each system both ZA and BC loops overlapped well between apo and holo BD structures, indicating that BD pocket made up of ZA and BC loops may be preorganized for RVX-208 binding. Then, the root-mean-square fluctuations (RMSFs) of Cα atoms of BD in apo and holo systems of BRD2-BD1 and BRD2-BD2 were calculated (Fig. 6). It is clear that, in either BD1 or BD2 system, high residue flexibility mainly occurs at three loop regions, namely ZA, AB and BC loop. Among them, ZA and BC loops participate in the interaction with RVX-208. Comparing RMSFs between apo and holo BDs in both systems, we found that BC loop almost kept unchangeable during the simulation, whereas the flexibility of ZA loop notably decreased upon binding to RVX-208. It is in accordance with the previously reported results of other inhibitor-BD systems^37, 38^. The higher flexibility decreasing of ZA loop of BD2 relative to BD1 indicated that BD2 pocket might undergo conformational rearrangement due to the binding of RVX-208. Additionally, we used the betweenness to characterize the residue-residue relevance in protein topology network. Figure 7 showed that the betweenness values of inhibitor-binding residues in BRD2-BD2 system were higher than that in BRD2-BD1 system especially for ZA loop, indicating that RVX-208 rearranged the binding pocket and made related residues more connected. Wilson *et al*.^39^ have found that the selectivity of a drug is dominated by the induced-fit process during the drug binding. Based on the above analyses, it can be seen that RVX-208 may initially bind to BD1/2 by conformational selection mechanism and preferentially interact with the preorganized pocket residues. It may be then accompanied by RVX-208-induced stabilization of ZA and BC loops via an induced fit in BRD2-BD2 system, resulting in the highly connected bromodomain. In this context, the steric barrier for movement away from the RVX-208-bound state may increase, effectively locking BD2 into a specific stable conformation. Therefore, RVX-208 selectivity is likely to result from ligand-induced structural stabilization by an induced fit mechanism.

**Figure 8:**
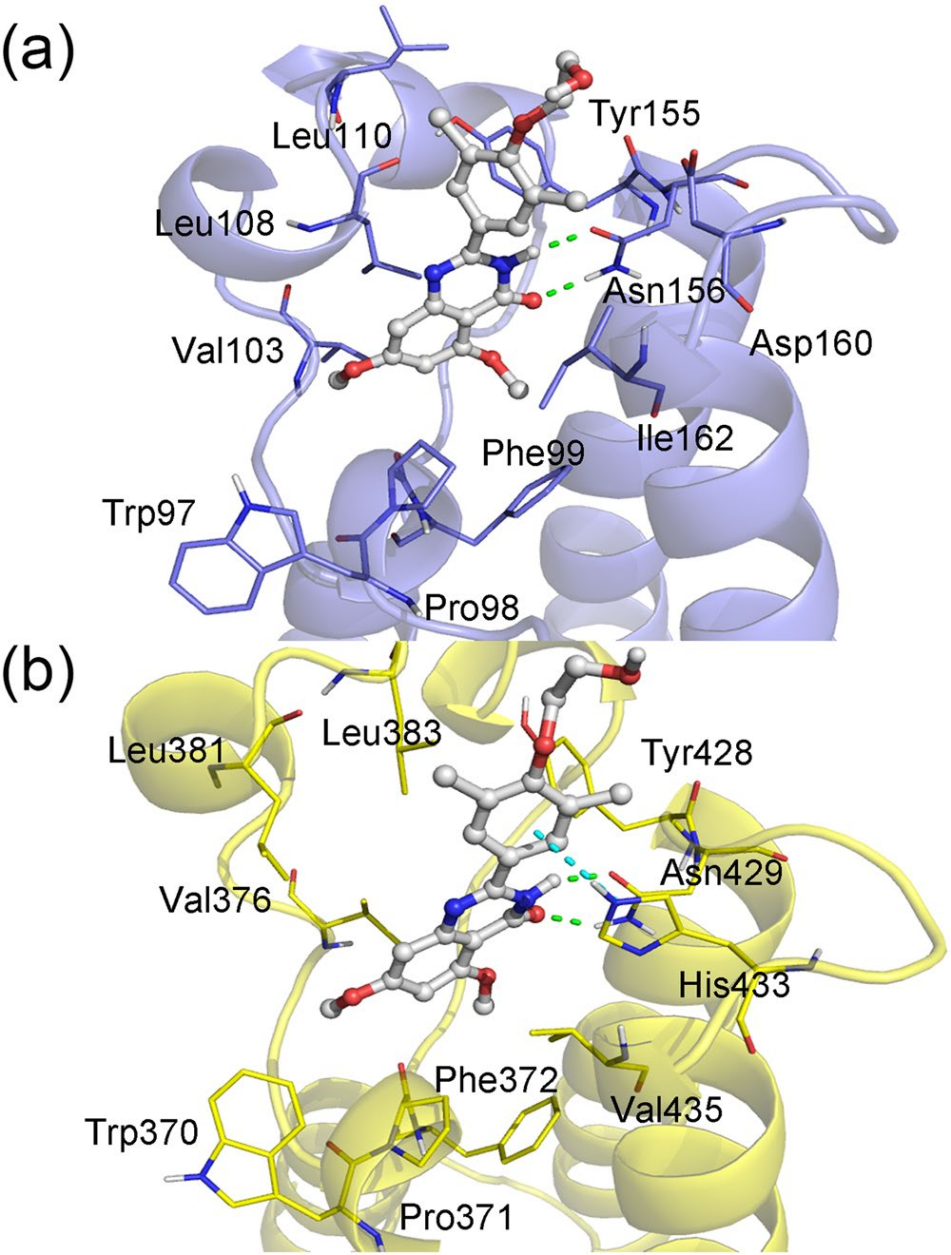
Binding mode of RVX-208 with (**a**) BRD2-BD1 and (**b**) BRD2-BD2. Green and blue dash lines are indicative of hydrogen bonds and π-π stacking interaction, respectively.

Next, we compared the detailed interactions of RVX-208 with BD1 and BD2. The representative conformation of each complex was extracted from their equilibrated trajectory by clustering analysis (Fig. 8). Due to high sequence homology of BD1 and BD2, the general binding mode of RVX-208 with BRD2-BD1 is similar to that with BRD2-BD2. Figure 8 shows that, except for His433 in BD2 system, the binding pocket is composed of hydrophobic residues, contributing to Δ*E*_vdw_ in Table 2. In combination with Fig. 3, two key residues, Leu110 and Asn156 (in BRD2-BD1 system), bind to RVX-208 mainly by hydrophobic and hydrogen bond (H-bond) interactions, respectively. The conserved Asn residue formed two strong H-bonds with the carbonyl oxygen and adjacent nitrogen of quinazolinone ring of RVX-208 which acted as an acetyl-lysine mimetic moiety. Figure 8 also showed that when binding to BDs, the hydroxyethoxy moiety of RVX-208 pointed out of pocket and stretched to the solvent, making only a few contacts with the bromodomain interface. By aligning representative structures of BD1 and BD2 systems, we found that this phenomenon was more notable in BRD2-BD2 system. Despite this, the previous study has showed that the lack of -O(CH_2_) _2_-OH moiety of RVX-208 could significantly affect its BD2 selectivity^21^. Hence, we expect that the sufficient contact of inhibitors to solvent at the above position is essential for its selectivity for BD2, and should be taken into much account in the design of selective inhibitors.

**Figure 9:**
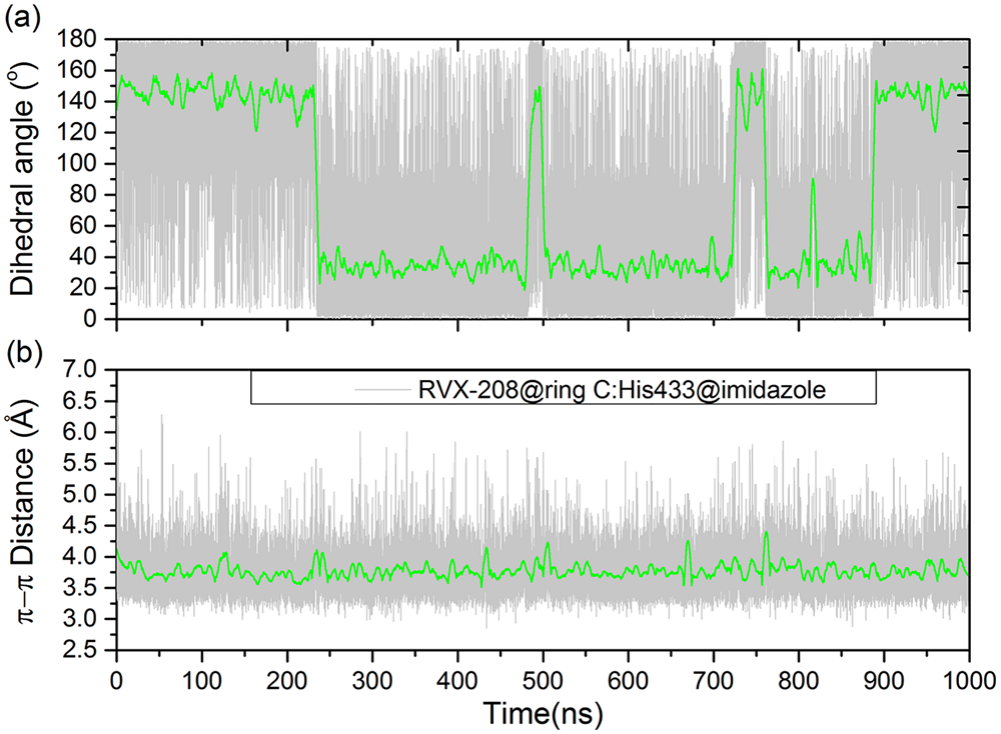
The monitored (**a**) dihedral angle and (**b**) distance between ring C of RVX-208 and imidazole ring of His433 of BRD2-BD2 during the simulation.

Actually, for the binding modes of RVX-208 and BD1/2, the largest differences were reflected from two residues, Ile162 (Val435 in BD2) and Asp160 (His433 in BD2) in BRD2-BD1 system, both of which were distinguishable residues between BD1 and BD2 (Table 1). Ile and Val residues are hydrophobic residues, interacting with RVX-208 mainly by hydrophobic interactions (Fig. 8). When binding to RVX-208, the energy contribution of Ile is lower than that of Val (Fig. 3). Ile or Val residue locates at the bottom of BD pocket, the relatively unfavorable contribution of isoleucine may derive from bigger volume of side chains, leading to that ring A of RVX-208 is not able to insert into deep pocket. As for Asp160 and His433, the former is unfavorable for binding to RVX-208 in BRD2-BD1 system, while the latter provides −0.66 kcal/mol contribution in BRD2-BD2 system (Fig. 3). As shown in Fig. 8a, Asp160 was far away from RVX-208, enlarging BD1 pocket to a certain extent. Nevertheless, when binding to RVX-208, the imidazole ring of His433 flips into pocket, packing against phenyl ring of RVX-208 and making the face-face aromatic interactions. Under this condition, BD2 pocket became more compact. A recent study based on RVX-297 (another BD2-selective inhibitor) has demonstrated that this kind of orientation of His433 could lead to a significant narrowing of BD2 pocket, then limiting the dimethyl-phenyl moiety in fixed conformation that is opposite to the free rotation as observed in BRD2-BD1 system^40^. The dihedral angle and distance between two aromatic rings of RVX-208 and His433 were then monitored over the simulation, respectively. Figure 9a showed that the angle of two aromatic rings fluctuated around 40° or 140°. The occurrence of complementary angle is mainly ascribed to the torsion of His433 imidazole ring. This is reasonable, considering the flexibility of His433 which locates at the loop domain and on protein surface (Fig. 8b). The distance of these two rings is stable at about 3.8 Å, keeping strong π-π interactions. Therefore, as the unique polar residue interacting with RVX-208, His433 is closely related to the rearrangement of BD2 pocket and crucial for preferentially binding to BD2.

Now, we have understood the molecular mechanism of selective inhibition of RVX-208 towards BD2. Then, several strategies for rational design of novel selective inhibitor of the second bromodomain were proposed. Firstly, the better shape complementarity and packing with BD2 are required for selective inhibitors. This derives from energy calculation result that van der Waals term is the dominant factor for RVX-208 selectivity. In particular, Asn429 (or Asn156), as the residue with largest contribution, can interact with RVX-208 by hydrophobic and H-bond interactions. Increasing the interactions between RVX-208 and Asn429 may shorten the shortest communication path between ZA and BC loops, and make BD2 pocket more compact. Furthermore, although the formed H-bonds of RVX-208 with Asn156 of BD1 and Asn429 of BD2 had minor difference, they were reported to be essential for the acetyl-lysine interaction of histones and involved in the binding to many other BET inhibitors^16, 41, 42^. Secondly, the hydrophilic group of inhibitors able to form enough contacts with the solvent is necessary for its selectivity. For instance, as to RVX-208, despite only a few contacts made between -O(CH_2_)_2_-OH group and BD2, the replacement of this group by -OH or -H can dramatically reduce its selectivity, as reported previously^21^. In our study, compared to BRD2-BD1 system, -O(CH_2_) _2_-OH of RVX-208 is more prone to point out to the solvent in BRD2-BD2 system. In addition, RVX-297, a compound found by Hansen *et al*.^40^, differs from RVX-208 only with the -O(CH_2_)_2_-OH replaced by -O(CH_2_)_2_-pyrrolidine. However, BD2-selectivity of the former increases by more than two times, further emphasizing the importance of hydrophilic groups in the structure of selective inhibitors. Thirdly, in the inhibitor structure, the functional group with large volume should not occur near Val435. The comparison analysis of Ile162 (BRD2-BD1) and Val435 (BRD2-BD2) shows that the large moiety may prevent inhibitor from entering into the bottom of BD2 pocket, thus disturbing the stable binding of protein and inhibitor. Finally, interactions between inhibitors and His433 need to be much considered. His433, a BD2-specific residue, has been reported to be closely related with RVX-208 preference for BD2 over BD1^21, 40^. Based on the above binding mode analysis, inhibitors should be designed close to His433 and the introduction of aromatic moiety facing the imidazole ring of His433 is highly recommended. The information gained from this study will provide valuable guidance for the identification and design of new inhibitors with improved binding properties and selectivity.

## Conclusions

In the present study, the long-time MD simulations were performed to study the inhibition and domain-selective mechanism of RVX-208 to BRD2-BD2 and BRD2-BD1. Results showed that the binding affinity between RVX-208 and BRD2-BD2 was strongest in three systems. Leu383 and Asn429 in BRD2-BD2 system (corresponding to Leu110 and Asn156 in BRD2-BD1 system) were the most key residues for binding to RVX-208. Compared to BRD2-BD1 system, RVX-208 can shorten the shortest communication path between ZA and BC loops of BD2, making pocket readjusted more suitable for RVX-208 binding. Val435 (Ile162 in BD1) and His433 (Asp160 in BD1), as the distinguishable residues of BD2 from BD1, play a key role for selective binding of RVX-208 to BD2. These results may help in fully understanding the selectivity mechanism for inhibition of BRD2-BD2 and -BD1, and guiding the future novel selective BD2 inhibitors.

## Material and Methods

### System preparations

Prior to simulation, we first need to obtain the complex structures of RVX-208 with BD1 and BD2. At present, crystal structures of RVX-208 in complex with BD1 of human BRD4 (PDB ID: 4MR4) and BD2 of human BRD2 (PDB ID: 4MR6) are both available from Protein Data Bank^43^. The high conservation of BD1s in BET family has been well known from previously studies^3, 5, 14^. However, to eliminate the influence brought by slight structural difference outside inhibitor pocket between BRD4 and BRD2, we also constructed complex structure of RVX-208 and BD1 of BRD2. Considering the fact that BD1 and BD2 are highly homologous and conservative in the binding site, structure-based alignment was used to guide the complex construction of RVX-208 and BD1 of BRD2. The protein in PDB 4MR4 (BRD4-BD1 and RVX-208) as a template, was aligned to target protein PDB 2YDW^41^ (BRD2-BD1 and GW841819X inhibitor). Then, the original GW841819X inhibitor was deleted, and RVX-208 extracted from template was merged into target protein. Three studied complexes were finally optimized by Prime and Protein Preparation Wizard in Schrodinger suite 2015^44^. To compare conformational changes of BDs induced by RVX-208’s binding, three apo-BD1 or -BD2 (without RVX-208) were also modeled.

### Molecular dynamics simulations

MD simulations were performed using GPU-accelerated PMEMD within AMBER14^45^. The AMBER ff14SB^46^ and general Amber force fields^47^ were used for protein and RVX-208, respectively. The inhibitor charges were assigned using restrained electrostatic potential partial charges. The atomic type/partial charge of RVX-208 were given in Table S1. Geometry optimization and electrostatic potential calculations were performed using Gaussian09^48^ at HF/6-31 G^*^ level. To neutralize net charges of each system, chlorine atoms were added as counter ions. TIP3P water molecules were added into the system, and the solute was at least 12 Å away from the boundary of water box. The prepared systems were minimized, heated and equilibrated. Then, 1 μs production run was carried out without any restraint in *NPT* ensemble. Temperature was regulated with Langevin thermostat using the “ig = −1” option to randomly set the random number seeds at each restart, avoiding synchronization effects. All the bonds involving hydrogen were constrained by SHAKE algorithm, and particle mesh ewald method^49^ was used to calculate long-range electrostatic interactions.

### Thermodynamic calculations

The binding free energy of RVX-208 and BRD2/4-BD1 or BRD2-BD2 was analyzed by both molecular mechanics generalized born surface area (MM-GBSA)^50, 51^ and molecular mechanics poisson boltzmann surface area (MM-PBSA)^52^ methods, integrated in AMBER14 package. Herein, a total of 1000 snapshots were extracted from the last equilibrated 200 ns trajectory with a time interval of 200 ps, and calculated:

1 $${\rm{\Delta }}G={G}_{{\rm{Complex}}}-({G}_{{\rm{BD}}}+{G}_{\mathrm{RVX}-\mathrm{208}})$$

2 $${G}_{{\rm{total}}}={E}_{{\rm{gas}}}+{G}_{{\rm{sol}}}-TS$$

3 $${E}_{{\rm{gas}}}={E}_{{\rm{vdw}}}+{E}_{{\rm{ele}}}$$

4 $${G}_{{\rm{sol}}}={G}_{\mathrm{sol}\_\mathrm{np}}+{G}_{\mathrm{sol}\_\mathrm{polar}}$$

5 $${G}_{\mathrm{sol}\_\mathrm{np}}=\gamma \cdot SASA$$

where *G*_complex_, *G*_BD_ and *G*_RVX-208_ are free energies of BD-RVX-208 complex, BD and RVX-208, respectively. *G* was estimated from gas-phase energy *E*_gas_ and solvation free energy *G*_sol_. *E*_gas_ contains an electrostatic term (*E*_ele_) and van der Waals term (*E*_vdw_). The solvation energy is further decomposed into polar (*G*_sol_polar_) and nonpolar solvation energies (*G*_sol_np_). The former was calculated by solving the generalized-born/poisson-voltzmann model. Dielectric constants for solute and solvent were set to 1 and 80, respectively. The latter was estimated by solvent accessible surface area (SASA) determined using a water probe radius of 1.4 Å. The surface tension constant *γ* was set to 0.0072 kcal/(mol·Å^2^)^53^. Entropic contributions (S) were estimated by NMODE module of AMBER14. Residue energy decomposition was also performed to identify the important contribution residues to the total binding free energy.

### Structural network analysis

The representative structures from clustering analysis with the last 200 ns trajectory were used to construct protein structural network. C_α_ atom of a residue is considered as a node, and a weighted edge is drawn if C_α_-C_α_ distance between a pair of residues is within a threshold distance, *R*_c_ (~7 Å). In our study, structural network was constructed by NAPS (network analysis of protein structures) platform^54^, which integrated the analysis and interactive visualization of protein contact networks. The shortest path distance between two nodes is the minimum number of nodes traversed to reach from one node to another, and displays the path of long-range interaction in the protein^55, 56^. The experimental study found that the residues in the shortest path could take part in the domain-domain communication and mediate signaling transfer^57^. Using Floyd-Warshall algorithm, two important residues, Leu110 from ZA loop and Asn156 from BC loop, were chosen as important residues to study the shortest communication path between these two loops. The betweenness of a node is defined as the number of shortest paths that pass through this node in the network, representing a global/local centrality of this node. MD simulations have elucidated the molecular determinant underlying ligand-induced modulation of conformational dynamics, showing that structural perturbations of high centrality sites by ligand binding may be coupled to conformational rearrangements of protein pocket or even long-range regulatory domain^55, 58^. Here, the betweenness parameter was used to characterize residue connectivity of apo and holo-BRD protein, especially for ZA and BC loops, which reflects the structural rearrangement of pocket induced by RVX-208.

## Electronic supplementary material

Supplementary information
