# Supplementary material for: Selective inhibition mechanism of RVX-208 to the second bromodomain of bromo and extraterminal proteins: insight from microsecond molecular dynamics simulations
Source: Sci Rep. 2017 Aug 18;7:8857. doi: 10.1038/s41598-017-08909-8 (PMC5562737; doi:10.1038/s41598-017-08909-8)
Supplement: Supplementary file 1 — Supplementary information [file 41598_2017_8909_MOESM1_ESM.doc]

**Selective inhibition mechanism of RVX-208 to the second bromodomain of bromo and extraterminal proteins: insight from microsecond molecular dynamics simulations**

Qianqian Wang^1^, Ying Li^1^, Jiahui Xu^1^, Yuwei Wang^1^, Elaine Lai-Han Leung^1*^ & Liang Liu^1*^, Xiaojun Yao^1*^

^1^State Key Laboratory of Quality Research in Chinese Medicine, Macau Institute for Applied Research in Medicine and Health, Macau University of Science and Technology, Taipa, Macau, China

^*^Corresponding authors

Tel.: +853-8897-2409

Fax: +853-2882-2799

E-mail address: lhleung@must.edu.mo; lliu@must.edu.mo; xjyao@must.edu.mo

**Table S1.** Atomic types and partial charges for RVX-208.


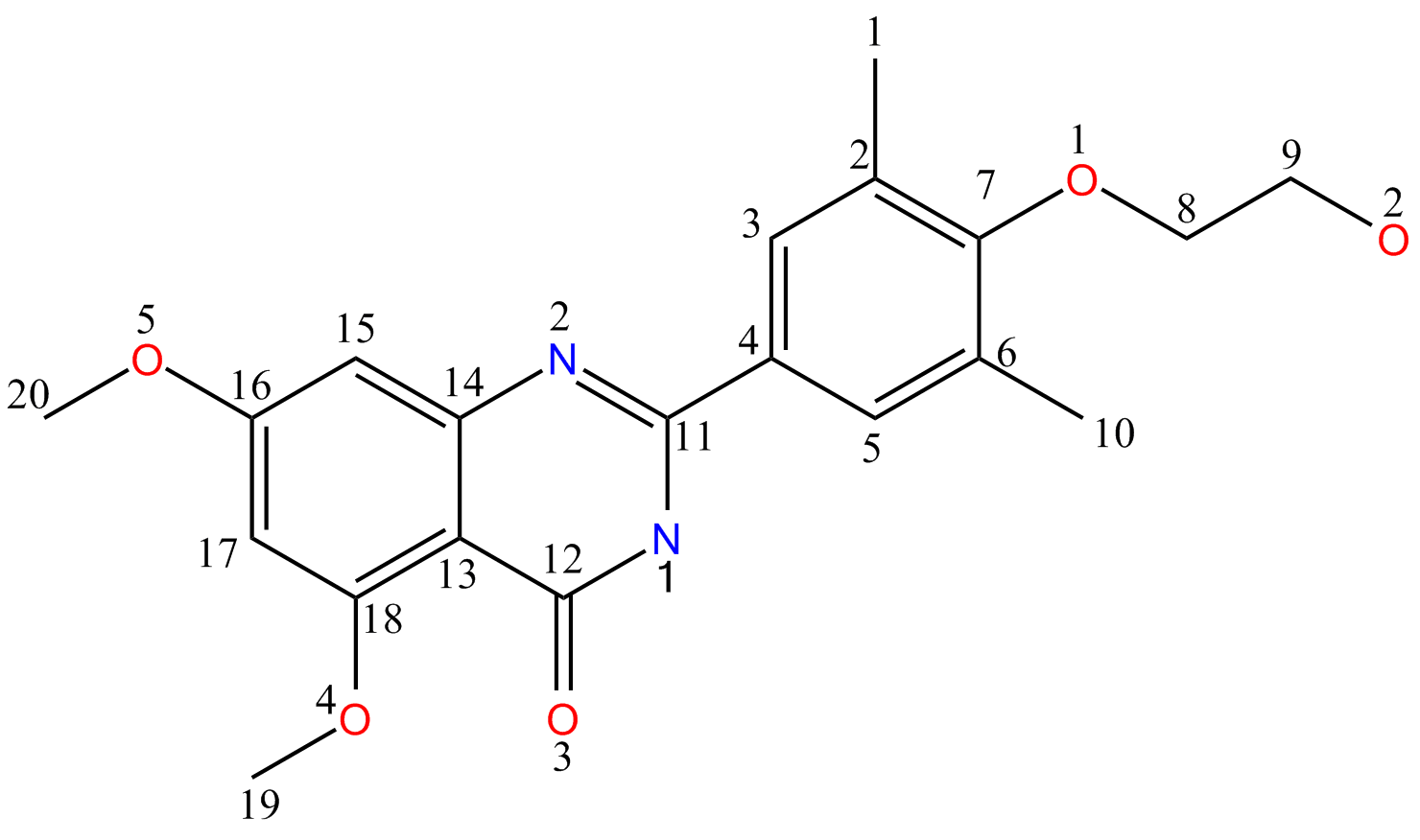


| **Atom name** | **Atom type** | **Partial charge** |
| --- | --- | --- |
| C1 | c3 | -0.398051 |
| C2 | ca | 0.345531 |
| C3 | ca | -0.407904 |
| C4 | ca | -0.074681 |
| C5 | ca | -0.407904 |
| C6 | ca | 0.345531 |
| C7 | ca | -0.102396 |
| O1 | os | -0.253447 |
| C8 | c3 | -0.052642 |
| C9 | c3 | 0.534895 |
| O2 | oh | -0.732787 |
| C10 | c3 | -0.398051 |
| C11 | cc | 0.844992 |
| N1 | n | -0.847467 |
| H1 | hn | 0.382437 |
| C12 | c | 1.051299 |
| O3 | o | -0.698826 |
| C13 | ca | -0.809937 |
| C14 | ca | 0.851349 |
| N2 | nd | -0.759663 |
| C15 | ca | -0.828325 |
| C16 | ca | 0.684344 |
| C17 | ca | -0.609389 |
| C18 | ca | 0.56503 |
| O4 | os | -0.217427 |
| C19 | c3 | -0.181135 |
| O5 | os | -0.357846 |
| C20 | c3 | -0.01754 |
| H2 | hc | 0.114784 |
| H3 | hc | 0.114784 |
| H4 | hc | 0.114784 |
| H5 | ha | 0.207435 |
| H6 | ha | 0.207435 |
| H7 | h1 | 0.07272 |
| H8 | h1 | 0.07272 |
| H9 | h1 | -0.076492 |
| H10 | h1 | -0.076492 |
| H11 | ho | 0.442382 |
| H12 | hc | 0.114784 |
| H13 | hc | 0.114784 |
| H14 | hc | 0.114784 |
| H15 | ha | 0.262373 |
| H16 | ha | 0.196965 |
| H17 | h1 | 0.110587 |
| H18 | h1 | 0.110587 |
| H19 | h1 | 0.110587 |
| H20 | h1 | 0.0735 |
| H21 | h1 | 0.0735 |
| H22 | h1 | 0.0735 |

**
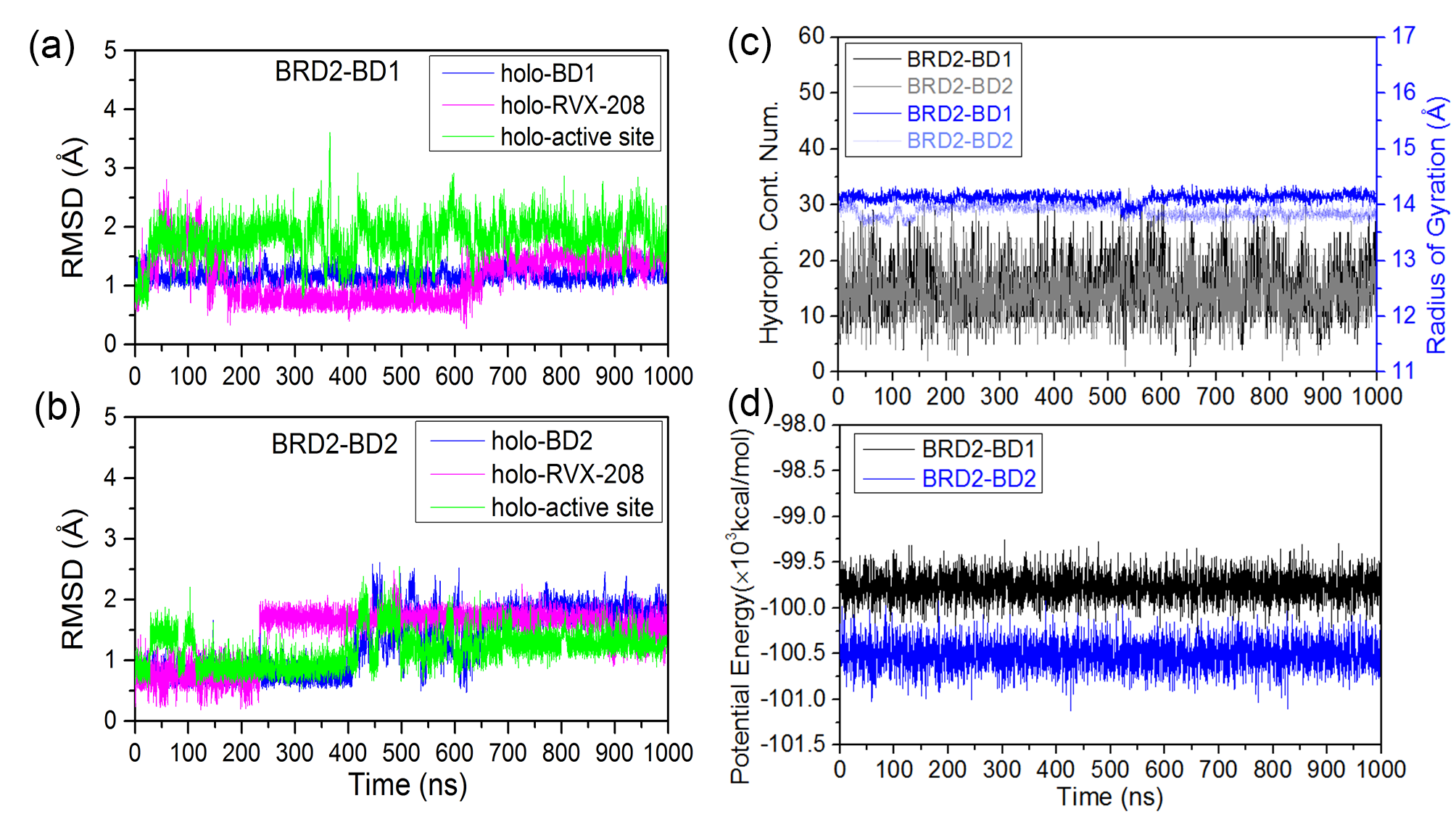
Figure S1.** Time series of RMSDs of protein, RVX-208 and active site in holo- (a) BRD2-BD1 and (b) BRD2-BD2 systems; time series of (c, left) contact number between CA atoms of hydrophobic residues of BDs and RVX-208, (c, right) radius of gyration of BDs and (d) potential energy for two complex systems in parallel trajectories.

**
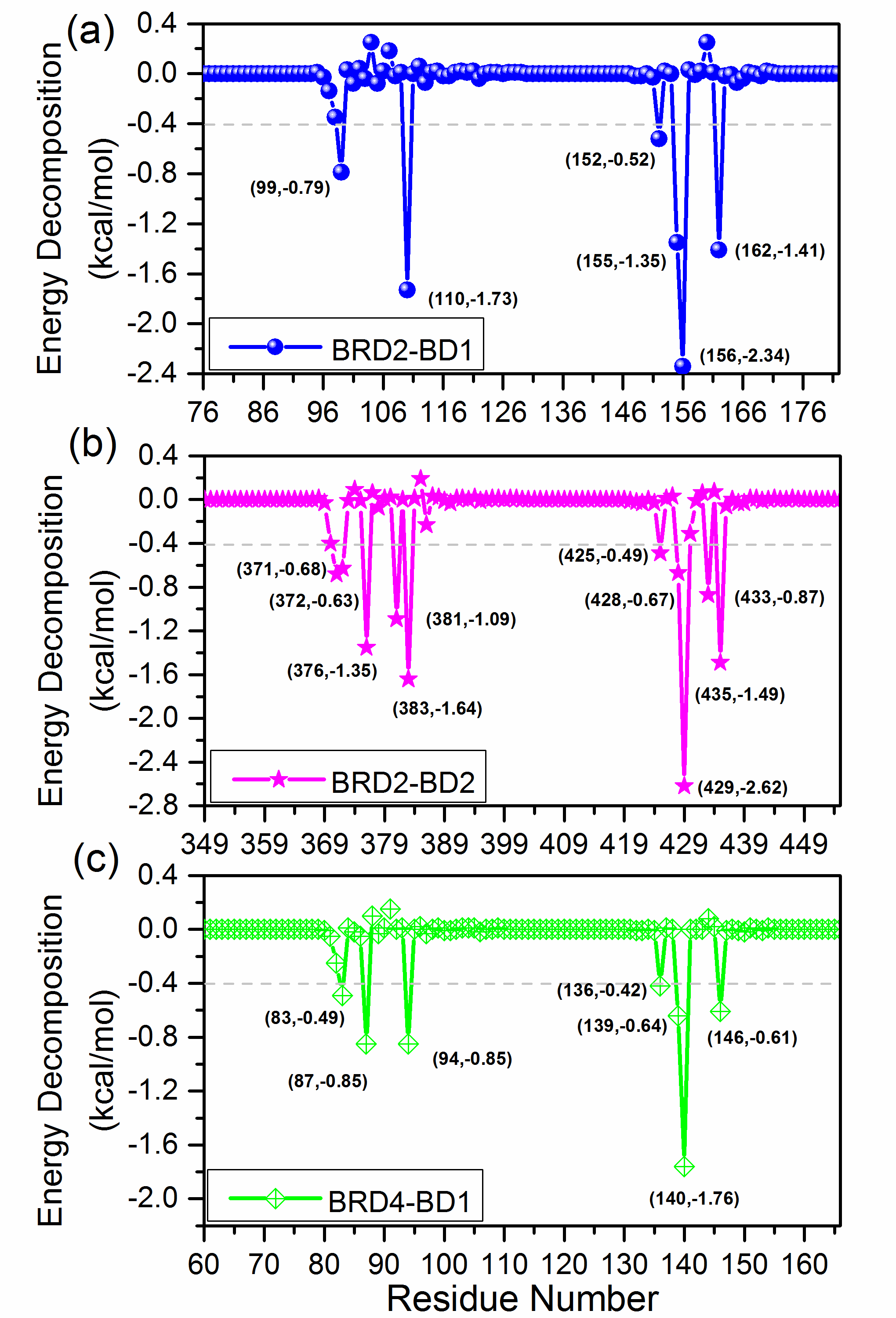
**

**Figure S2.** Per-residue energy decomposition in (a) BRD2-BD1, (b) BRD2-BD2 and (c) BRD4-BD1 systems in the parallel trajectories. Residues with >0.4 kcal/mol contribution were labeled.

**
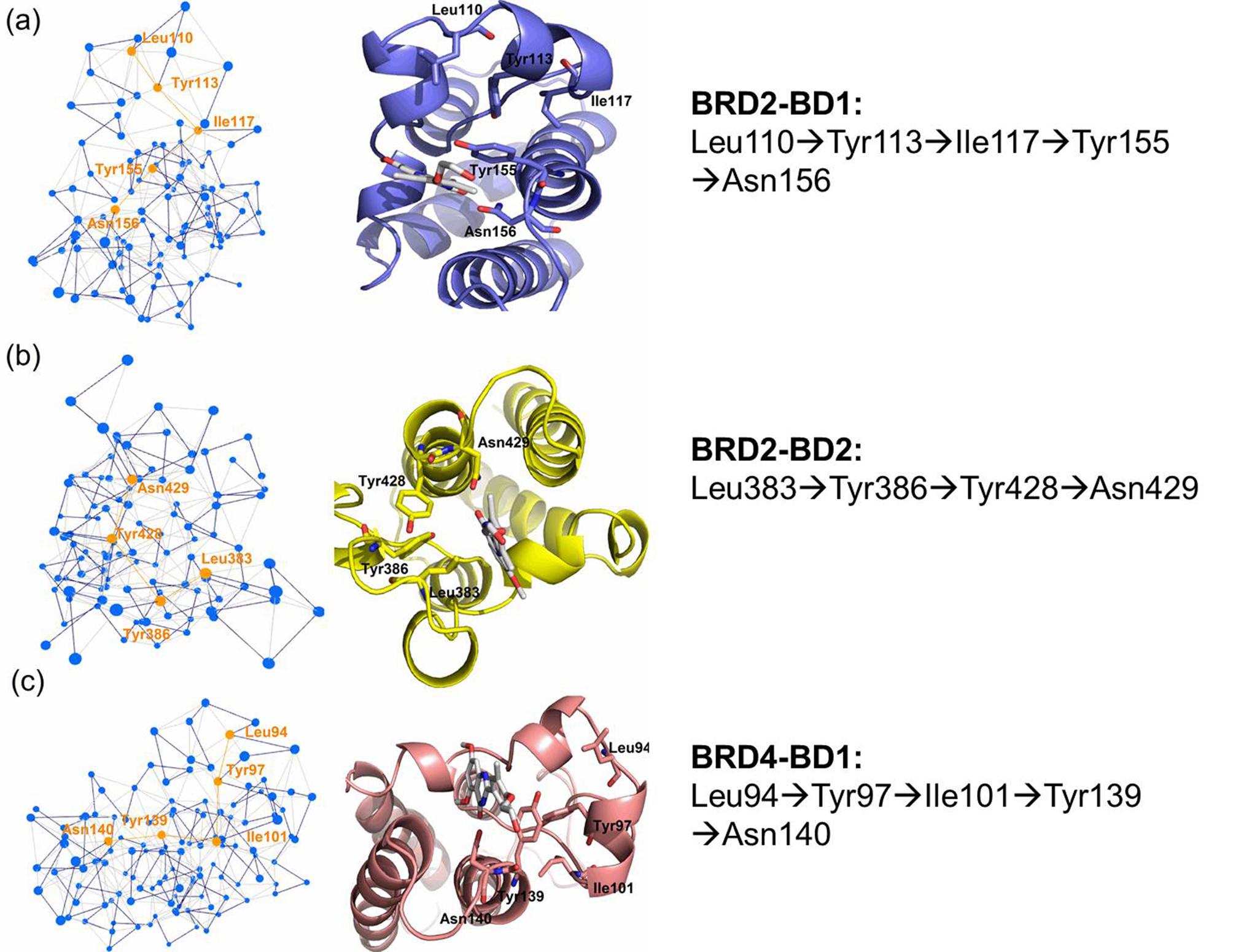
**

**Figure S3.** Shortest communication path between ZA and BC loops and the corresponding structure in (a) BRD2-BD1, (b) BRD2-BD2 and (c) BRD4-BD1 systems in the parallel trajectories.
